# UNCOVER registry: A searchable online catalogue for COVID-19 evidence reviews

**DOI:** 10.7189/jogh.10.020101

**Published:** 2020-12

**Authors:** Wei Xu, Xiaomeng Zhang, Yazhou He, Marshall Dozier, Brendan Owers, Xue Li, Evropi Theodoratou, on behalf of UNCOVER

**Affiliations:** 1 Centre for Global Health, Usher Institute, University of Edinburgh, Edinburgh, UK; 2 College of Medicine and Veterinary Medicine, University of Edinburgh, Edinburgh, UK; 3 School of Public Health, Zhejiang University, Hangzhou, China; 4 Cancer Research UK Edinburgh Centre, Medical Research Council Institute of Genetics and Molecular Medicine, University of Edinburgh, Edinburgh, UK

## COVID-19 PANDEMIC AND LITERATURE EXPLOSION

An epidemic of novel Coronavirus Disease 2019 (COVID-19) causing an acute respiratory disease began in December 2019, triggered a Public Health Emergency of International Concern (PHEIC) and was declared a pandemic on 11 March 2020 by the World Health Organisation (WHO). Globally, there has been an accumulated number of 7 823 289 confirmed cases and 431 541 deaths occurred in 216 countries/territories according to the report of WHO of 15 June 2020 [1].

An unprecedented amount of research has been conducted on all aspects of COVID-19, and the academic and clinical communities are facing a rapidly growing body of outputs. Systematic and standardised evidence collection and synthesis on a large scale is pivotal. These findings can accelerate our understanding of COVID-19 and facilitate informed decision-making in the response to the pandemic.

An online collection or registry of published or ongoing reviews specific to COVID-19 literature can be utilised to promote transparency of reporting, to avoid duplicate efforts, to assist evidence triangulation and to guide policy and/ or clinical decision making [2]. During the ongoing pandemic, organisations including the WHO [3], the Cochrane network [4], the Centre for Evidence-Based Medicine (CEBM) [5], and the European Centre for Disease Prevention and Control (ECDC) [6] have built and maintain comprehensive data repositories to gather all publications related to COVID-19. In addition to these repositories, there has also been an urgent need to develop a registry of all conducted or on-going ***COVID-19 evidence reviews*** to provide a landscape on research topics that either have been or are being reviewed.

The UNCOVER (Usher Network for COVID-19 Evidence Reviews) group from The University of Edinburgh launched a register of conducted or ongoing reviews on 24 March 2020 (https://www.ed.ac.uk/usher/uncover/register-of-reviews). UNCOVER is a network of population health researchers and information specialists who are committed to responding quickly to requests from policymakers for evidence reviews. UNCOVER registry aims to index all the reviews available online that summarised epidemiological evidence on COVID-19 for policymakers, clinicians, and researchers in response to the COVID-19 outbreak.

## HOW UNCOVER REGISTRY WORKS

### Registry website

The University of Edinburgh web team weekly updates the UNCOVER registry (please visit the following link: https://www.ed.ac.uk/usher/uncover/register-of-reviews). COVID-19 evidence reviews can be filtered by review type, language, publication time, and/or use search terms (or key concepts) through the search box. A csv file of the complete registry can also be downloaded. Title, publication date, language, database and DOI are displayed directly in the list of searching results; abstracts can be checked through dropdown buttons. We recommend that researchers consult this register before embarking on a new review, to minimize duplication of effort. In addition, a step-by-step guide for conducting COVID-19 rapid evidence reviews using the UNCOVER methodology is available on this site.

### Maintenance and updating of the registry

We run a systematic search weekly in PubMed, medRxiv and WHO COVID-19 database since 24 March 2020, and we manually screen CEBM, ECDC, Imperial College London [7] websites, to collate all COVID-19 evidence reviews (please see search strategies in **Table 1**). We review titles, abstracts and subsequently full texts to identify publications based on predefined inclusion and exclusion criteria. In particular, we include 1) all COVID-19 evidence reviews (publication types include scoping reviews, systematic reviews and meta-analyses, rapid reviews, narrative reviews) or protocols of evidence reviews; 2) reviews in all languages; 3) reviews in peer-reviewed journals and pre-prints. Conversely, we exclude personal opinions published as commentaries, conference abstracts, interviews and editorials.

**Table 1:** Search strategies

Databases	Detailed search strategies
PubMed	((“systematic review”[Publication Type] OR “meta-analysis”[Publication Type] OR “rapid review”[Title/Abstract] OR “systematic review”[Title/Abstract] OR “scoping review”[Title/Abstract] OR “meta-analysis”[Title/Abstract])
	AND
	(“Betacoronavirus”[Mesh] OR “Coronavirus Infections”[MH] OR “Spike Glycoprotein, COVID-19 Virus”[NM] OR “COVID-19”[NM] OR “Coronavirus”[MH] OR “Severe Acute Respiratory Syndrome Coronavirus 2”[NM] OR 2019nCoV[ALL] OR Betacoronavirus*[ALL] OR Corona Virus*[ALL] OR Coronavirus*[ALL] OR Coronovirus*[ALL] OR CoV[ALL] OR CoV2[ALL] OR COVID[ALL] OR COVID19[ALL] OR COVID-19[ALL] OR HCoV-19[ALL] OR nCoV[ALL] OR “SARS CoV 2”[ALL] OR SARS2[ALL] OR SARSCoV[ALL] OR SARS-CoV[ALL] OR SARS-CoV-2[ALL] OR Severe Acute Respiratory Syndrome CoV*[ALL]))
**WHO database**	(advanced search with entry date limits adjusted as needed)
	(tw:(“systematic review” OR “rapid review” OR “meta analysis” OR meta-analysis)) AND (tw:(entry_date:(2020032* OR 202004* OR 202005* OR 202006*)))
**medRxiv**	(advanced search)
	Covid terms cluster combined with OR:
	COVID-19
	[Cc]oronavirus
	SARS-CoV-2
	2019-nCoV
	Review terms cluster combined with OR:
	[Ss]ystematic review
	[Rr]apid review
	[Mm]eta analysis
	[Mm]eta-analysis
	[Mm]etaanalysis
**Other Websites (CEBM, ECDC, ICL)**	No available search filters. Manual search by browsing the list of publications

For each included review, the following items are extracted and recorded: database, type of work, title, author, abstract, year, journal/source, volume, number, pages, date, URL, DOI, keywords, language. Extraction is conducted by three reviewers (WX, XZ, YH) and checked by a senior epidemiologist (ET).

### Snapshot of registry for week commencing 15 June 2020

**Figure 1:**
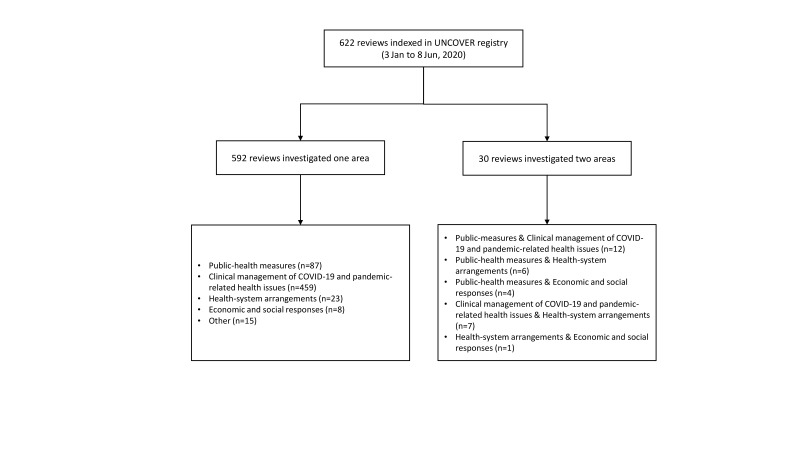
A categorical summary of the indexed reviews in UNCOVER registry.

A total of 622 reviews including 396 systematic reviews and meta-analyses, 193 rapid reviews, 23 scoping reviews, two umbrella reviews, one narrative review and seven protocols have been indexed in the registry (From 3 Jan to 8 Jun, 2020). All indexed reviews were categorised thematically according to McMaster University COVIDEND (**COVID**-19 **E**vidence **N**etwork to support **D**ecision-making) taxonomy [8]. A total of 592 reviews investigated one area: Public-health measures (n = 87), Clinical management of COVID-19 and pandemic-related health issues (n = 459), Health-system arrangements (n = 23), Economic and social responses (n = 8), Other (n = 15); A total of 30 reviews investigated two areas: Public-measures & Clinical management of COVID-19 and pandemic-related health issues (n = 12), Public-health measures & Health-system arrangements (n = 6), Public-health measures & Economic and social responses (n = 4), Clinical management of COVID-19 and pandemic-related health issues & Health-system arrangements (n = 7), Health-system arrangements & Economic and social responses (n = 1) (Please see **Figure 1**).

The UNCOVER group will continue to promote systematic and transparent COVID-19 evidence research and conduct evidence grading assessment to provide the most reliable research evidence for scientists, clinical professionals and policymakers.

## References

[1] World Health Organization. Coronavirus disease (COVID-2019) situation reports. 2020. Available on: https://www.who.int/docs/default-source/coronaviruse/situation-reports/20200615-covid-19-sitrep-147.pdf?sfvrsn=2497a605_4. Accessed: 16 June 2020.

[2] Stewart L Moher D Shekelle P Why prospective registration of systematic reviews makes sense. Syst Rev. 2012;1. 10.1186/2046-4053-1-7 22588008 PMC3369816

[3] World Health Organization. COVID-19 Global literature on coronavirus disease. Available: https://search.bvsalud.org/global-literature-on-novel-coronavirus-2019-ncov/. Accessed: 9 June 2020.

[4] Cochrane Library. Coronavirus (COVID-19). Available: https://www.cochranelibrary.com/covid-19. Accessed: 9 June 2020.

[5] Oxford COVID-19 Evidence Service - CEBMAvailable: https://www.cebm.net/covid-19/. Accessed: 9 June 2020.

[6] European Centre for Disease Prevention and Control. COVID-19 pandemic. Available: https://www.ecdc.europa.eu/en/covid-19-pandemic. Accessed: 9 June 2020.

[7] Imperial College London. COVID-19. Available: https://www.imperial.ac.uk/medicine/departments/school-public-health/infectious-disease-epidemiology/mrc-global-infectious-disease-analysis/covid-19/?fbclid=IwAR0xGvI2r5HC0LAURNShDjIJ_kwc_efgBYFwuFDt5GekAGJ80G3GVr7t268. Accessed: 9 June 2020.

[8] McMaster University. COVID-END. Available: https://www.mcmasterforum.org/networks/covid-end/resources-to-support-decision-makers/taxonomy-of-decisions/context. Accessed: 10 June 2020.

